# Regulatory Networks Involving STATs, IRFs, and NFκB in Inflammation

**DOI:** 10.3389/fimmu.2018.02542

**Published:** 2018-11-13

**Authors:** Ekaterini Platanitis, Thomas Decker

**Affiliations:** Max F. Perutz Laboratories, Department of Microbiology, Immunobiology and Genetics, University of Vienna, Vienna, Austria

**Keywords:** inflammation, macrophage, transcription, STAT, IRF, NFκB, epigenetic, chromatin

## Abstract

Cells engaging in inflammation undergo drastic changes of their transcriptomes. In order to tailor these alterations in gene expression to the requirements of the inflammatory process, tight and coordinate regulation of gene expression by environmental cues, microbial or danger-associated molecules or cytokines, are mandatory. The transcriptional response is set off by signal-regulated transcription factors (SRTFs) at the receiving end of pathways originating at pattern recognition- and cytokine receptors. These interact with a genome that has been set for an appropriate response by prior activity of pioneer or lineage determining transcription factors (LDTFs). The same types of transcription factors are also critical determinants of the changes in chromatin landscapes and transcriptomes that specify potential consequences of inflammation: tissue repair, training, and tolerance. Here we focus on the role of three families of SRTFs in inflammation and its sequels: signal transducers and activators of transcription (STATs), interferon regulatory factors (IRFs), and nuclear factor κB (NFκB). We describe recent findings about their interactions and about their networking with LDTFs. Our aim is to provide a snapshot of a highly dynamic research area.

## Introduction

Inflammation is a rapid response of the innate immune system to infection or sterile causes of trauma and tissue damage. Its main purpose is to alert, recruit, and activate cells of the immune system, mobilize the adaptive immune system, remove the infectious agent or other proinflammatory stimuli and, ultimately, repair the tissue damage inflicted by both the trigger of inflammation and the inflammatory process (1). These events require the coordinate action of a multitude of different cell types of the immune system and the inflamed tissue. Inflammation ensues when cells sense microorganisms by means of microbe-associated molecular patterns (MAMPs) or damaged tissue by the release of damage (or danger)- associated molecular patterns (DAMPs). Both MAMPs and DAMPs are recognized by binding to one or more pattern recognition receptors (PRR). Signal transduction by these receptors enables cells to mobilize a proinflammatory gene expression program (2, 3). As a corollary, antimicrobial effector mechanisms are activated and immune mediators are released that prepare the surrounding tissue for inflammation, cause influx of leukocytes from the blood and allow for the recruited cells to adopt an immunologically activated state.

The progression to a proinflammatory state necessitates dramatic transcriptome changes of the participating cells. Cells such as macrophages with a pivotal role in orchestrating inflammation acquire an appropriately structured genome during differentiation (4). In molecular terms this means that their chromatin ensures accessibility of critical regulatory DNA, thus allowing for an immediate transcriptional response of proinflammatory genes. Cell lineage specificity of genome accessibility requires a compatible (lack of) genome compaction and 3D structure, but also the activity of lineage-determining transcription factors (LDTFs). LTDFs belong to the larger group of pioneer transcription factors with the ability to bind enhancer elements within nucleosomal DNA. Their association with DNA causes nucleosome rearrangement and, at neighboring histones, the deposition of posttranslational modifications (marks) characteristic of accessible or poised enhancers. To initiate a proinflammatory response, MAMPs, DAMPs, or activating cytokines such as interferon γ (IFNγ) cause the synthesis and activation of signal-regulated transcription factors (SRTFs) that interact with the prearranged genome to cause a stimulus and cell-type specific transcriptome change (5). Likewise, suppression and resolution of inflammation and tissue repair result from signals targeting a different set of SRTFs to produce an anti-inflammatory response tailored to a particular cell-lineage by a permissive genome structure.

Signal transducers and activators of transcription (STATs), interferon regulatory factors (IRFs) and nuclear factor κB (NFκB) are major players among SRTFs. Different family members function in the establishment as well as the resolution or prevention of inflammation. This dual mode of macrophage activity during inflammation is represented by the polarization of macrophages into the proinflammatory M1 type and the inflammation-resolving M2 type. While these types, generated *in vitro* by using IFNγ/LPS (M1) or IL4 (M2), represent extremes with most likely no direct *in-vivo* equivalent (6–9), they establish a useful heuristic concept that has produced much insight how macrophages realize their pro- and anti-inflammatory potential.

## STATs, IRFs, and NFκB, a brief overview

For in-depth information and additional references concerning these transcription factor families the reader is referred to comprehensive reviews (10–15).

### STATs

**Figure 1:**
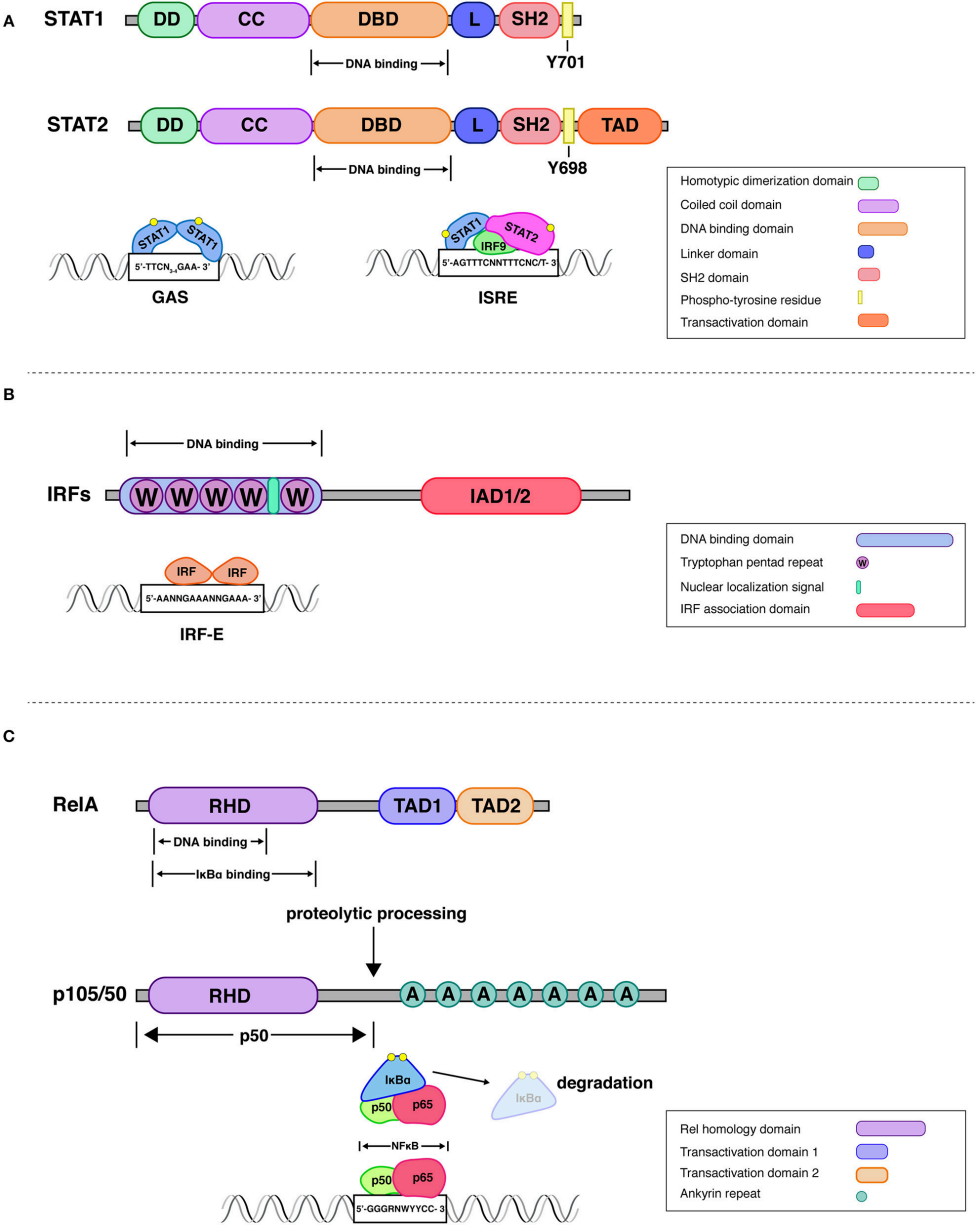
Structural attributes of STAT-, IRF-, and Rel family transcription factors. **STATs**. All mammalian STAT members share a common structural motif consisting of an N-terminal domain, which plays a role in dimerization (DD), followed by a coiled-coil domain (CC), that can be involved in interactions with other proteins, a DNA-binding domain (DBD), a linker domain (L), an SH2 domain for reciprocal phospho-tyrosine interaction and a transactivation domain (10, 11). Upon receptor engagement Janus kinases lead to the activation of the latent cytoplasmic STATs, via phosphorylation on single tyrosine residues (Y701 on STAT1 and Y690 on STAT2). The STAT1-STAT2 dimer associates with interferon regulatory factor 9 (IRF9) to form a transcriptionally active IFN-stimulated gene factor 3 (ISGF3). This complex controls gene expression by binding to interferon-stimulated response elements (ISRE) present in promoters of IFN stimulated gene (ISG). Additionally, STAT1 homodimers, translocate to the nucleus and stimulate ISG expression by binding to gamma interferon-activated sites (GAS) (17). **IRFs**. All IRFs harbor a conserved N-terminal DNA-binding domain (DBD), which forms a helix-turn-helix domain with a conserved tryptophan cluster that recognizes DNA sequences in interferon induced genes (18). An analysis of the crystal structure of the DBD of IRF1 bound to the Ifnb promoter revealed that 5′-GAAA-3′ is the consensus sequence recognized by the helix-turn-helix motif of IRF1 (19). This DNA motif is known as the IRF-element (IRF-E) (20). All IRFs harbor a C-terminal IRF association domain (IAD), which is responsible for homo- and heteromeric interactions with other family members or transcription factors (21, 22). IAD1 and IAD2 domains can be distinguished by structural criteria and are found, respectively, in IRF1 and IRF2 or all other IRFs. **Rel (NF**κ**B)**. One of the best studied NFκB dimers is the p50/p65 heterodimer, whose crystal structure has been solved (23). NFκB recognizes 9–11 bp (base pair) DNA-elements, which are often located within promoters and enhancers of NFκB target genes. The consensus sequence 5′-GGGRNWYYCC-3′, where R denotes a purine base, N means any base, W stands for adenine or thymine and Y represents a pyrimidine base, is recognized by the Rel-homology domain [RHD; (12)]. The C-terminal domain of RelA (p65) contains two strong and independent transactivation domains (TAD) providing full transcriptional activity (24). The p100 precursor protein is proteolytically processed to the NFκB subunit p50. The mature p50 protein contains the RHD followed by glycine-rich region, a region that is essential for directing the cleavage and proteolytic processing of a long IκB-like C-terminal part of the precursors (25). IκBα regulates rapid and transient induction of NFκB activity. The crystal structure of IκBα bound to the p65/p50 heterodimer revealed that one IκBα molecule binds to an NFκB dimer and masks the NLS of p65. IKKβ is necessary and sufficient for phosphorylation of IκBα, leading to IκBα ubiquitination, and further degradation by the proteasome.

STATs form a family of 7 members (STATs 1-4, STATs 5a and 5b, STAT6). All family members function predominantly in the context of cytokine-responsive, two-component JAK-STAT pathways. When cytokine receptors bind their cognate ligands, one or more receptor-associated Janus protein tyrosine kinases (JAKs) are activated and phosphorylate latent STATs on a single tyrosine residue. In some STATs this leads to a reorientation of preformed dimers into a parallel arrangement (16) whereas others may dimerize *de-novo*. Dimerization is stabilized by reciprocal phosphotyrosine (pY) interactions with SH2 domains which are, among transcription factors, a distinguishing feature of STATs (Figure 1). pY-mediated dimerization exposes an unconventional nuclear localization signal that shifts the subcellular localization of STATs to the nucleus (26). Homo-or heterodimeric STATs recognize a DNA sequence called gamma-interferon-activated sequence [GAS, TTCN_3−4_GAA; (17)]. In contrast, STATs able to form a complex with a non-STAT subunit, IRF9, bind to a distinct sequence, the interferon-stimulated response element [ISRE; (27)]. The ISRE is a hallmark of all type I and type III IFN-responsive genes (ISG) and a large fraction of IFNγ-inducible genes. Both type I IFN (IFN-I: IFNα, IFNβ, others) and type III IFN (IFN-III: IFNλ) cause tyrosine phosphorylation of STAT1 and STAT2, the resulting heterodimer forms an ISRE-dependent complex with IRF9 called ISGF3. As an addendum to this original paradigm of IFN-I signaling, research in recent years has produced a variety of activities of IRF9-containing complexes other than ISGF3 and of unphosphorylated STATs (U-STATs), both as transcriptional regulators and in non-nuclear contexts (28–30). We and others have recently reviewed these non-canonical STAT activities (31–33). Unlike IFN-I and IFN-III, the IFNγ-stimulated JAK-STAT pathway produces a STAT1 dimer, the gamma-interferon-activated factor (GAF), in addition to a very low level of ISGF3. This difference in STAT activation is one of the factors responsible for overlapping, yet discrete IFN-I and IFNγ-induced transcriptomes (29).

### IRFs

The original description and eponymous function of IRFs derives from their activity as regulators of the genes encoding type I IFN (34). The minimal IRF binding site of the IFNβ promoter, characterized by 5′-GAAA-3′ motifs (35) is part of many ISRE sequences (5′-PuPuAAANNGAAAPyPy-3′). Not surprisingly therefore, a second identification of IRFs resulted from the purification of ISRE-associated proteins (36). Subsequent work revealed a total of 9 family members in mice and men (IRF1-9). Common structural features of IRFs include an N-terminal DNA-binding domain with 5 characteristically spaced tryptophanes and one of two structurally distinct C-terminal IRF association domains (IAD; Figure 1). In IRF9, but not other IRFs, the IAD contains a binding site for STAT2 (37). The regulation of IRF transcriptional activity requires for some IRFs (IRF3, IRF5, IRF7) the phosphorylation of serine residues within the IAD and C-terminus for dimerization and activation. For others (IRF1, IRF2, IRF4, IRF8), sporadic reports of phosphorylation events exist (38, 39), but this modification is most likely not generally necessary to regulate transcriptional activity. IRF9 is unique as it has no known transcriptional activity on its own and is so far characterized exclusively as a subunit of ISGF3 or other complexes containing either STAT1 or STAT2.

Phosphorylation-dependent IRFs function downstream of toll-like receptors (TLR) and cytosolic nucleic acid receptors (2, 3). The established serine kinases are IKKβ [IRF5; (40)] and the non-canonical IKKs TBK1 and IKKε (41). IRF3 and IRF7 are the main regulators of IFN-I gene expression, whereas IRF5's main activity appears to be in the regulation of typical proinflammatory genes [although IRF5 may in some situations also contribute to IFN-I regulation; (42)]. With the notable exception of IRF3 all IRF family members are cytokine-inducible and have important functions in cytokine responses. Particularly IRF4 and IRF8 are important for the specification of immune cell lineages and/or as determinants of their functional attributes. IRF6 is the only family member with a function in embryonic development.

### NFκB

Various canonical and non-canonical NFκB complexes are formed by members of the Rel family of transcription factors which contain a Rel homology domain (RHD) for DNA binding and, in case of the RelA, RelB, and c-Rel family members, a C-terminal transactivation domain (12). The p52 and p50 proteins are formed from larger precursor protein (p100 and p105, respectively) by proteolytic processing. The major player among proinflammatory NFκBs, and the only one discussed here, is the heterodimer of RelA/p65 and p50. Its nuclear activity is restricted by inhibitors of NFκB (IκB, mainly IκBα) through a direct interaction that masks the nuclear localization signal. Activation of the IκB kinase complex (IKK complex, consisting of IKKα, IKKβ, and IKKγ/NEMO) causes the phosphorylation of IkBα on two critical serines which leads to its ubiquitination and subsequent degradation. In the innate immune system activation of the IKK complex is caused by all PRR, and by TNF receptor- as well as CD40-related receptor families. In the adaptive immune system NFκB is activated by these same receptor families and also by lymphocyte antigen receptors.

## Transcription factor networks involving STATs, IRFs, and NFκB in macrophages

There are several conceptual possibilities how transcription factors form networks (Table 1). Networking may result from a common functional context such as inflammation, but result from independent action. On the other hand, there are several ways to directly link the activity of one transcription factor to the activity of the other. For example, networking transcription factors may regulate each other's synthesis or activation or, alternatively, converge at promoter level to cooperate or antagonize each other in the regulation of a common set of genes. Such agonistic or antagonistic action can result from direct physical contacts or from complementing each other in different steps of promoter activation such as chromatin remodeling and modification or the formation of a transcriptional initiation complex. In one way or another all these possibilities contribute to networks containing STATs, IRFs, and NFκB.

**Table 1:** Different molecular principles guiding the interaction between transcription factors (TFs) of the STAT, IRF and Rel/NFκB families.

**Mode of interaction**	**Example**	**References**
TF1 regulates TF2 synthesis	•STAT1 regulates IRF1 and IRF8 synthesis •NFκB regulates IRF1 synthesis	(43) (44) (45)
Promoter occupancy of TF1 required for binding of TF2	•IRF8 required for NFκB/IRF3/7-dependent Ifnb enhanceosome assembly by LPS •IRF8 enhances IFNγ-induced gene transcription by STAT1 and IRF1 in myeloid cells	(46) (47) (48)
Promoter co-occupancy by TFs required for transcriptional activation	•ISGF3 and NFκB cooperate at iNOS and IL6 promoters •STAT1 and IRF1 cooperate in IFNγ-induced transcription	(49) (50) (51) (52)
Physical interaction of TFs	•IRF9 binds to STAT2 •IRF3 associates with RelA/p65 to function as coactivator at NFκB sites. Conversely, RelA/p65 functions as IRF3 coactivator at ISREs •U-STAT2 associates with RelA/p65 to induce IL6 transcription	(10) (37) (53) (54) (55)
TF competition for promoter binding (direct or indirect)	•STAT6 prevents NFκB binding at overlapping sites •STAT5 prevents STAT1 association at IRF8 promoter	(56) (57) (58)

### Establishment of enhancers controlling inflammatory gene expression

**Figure 2:**
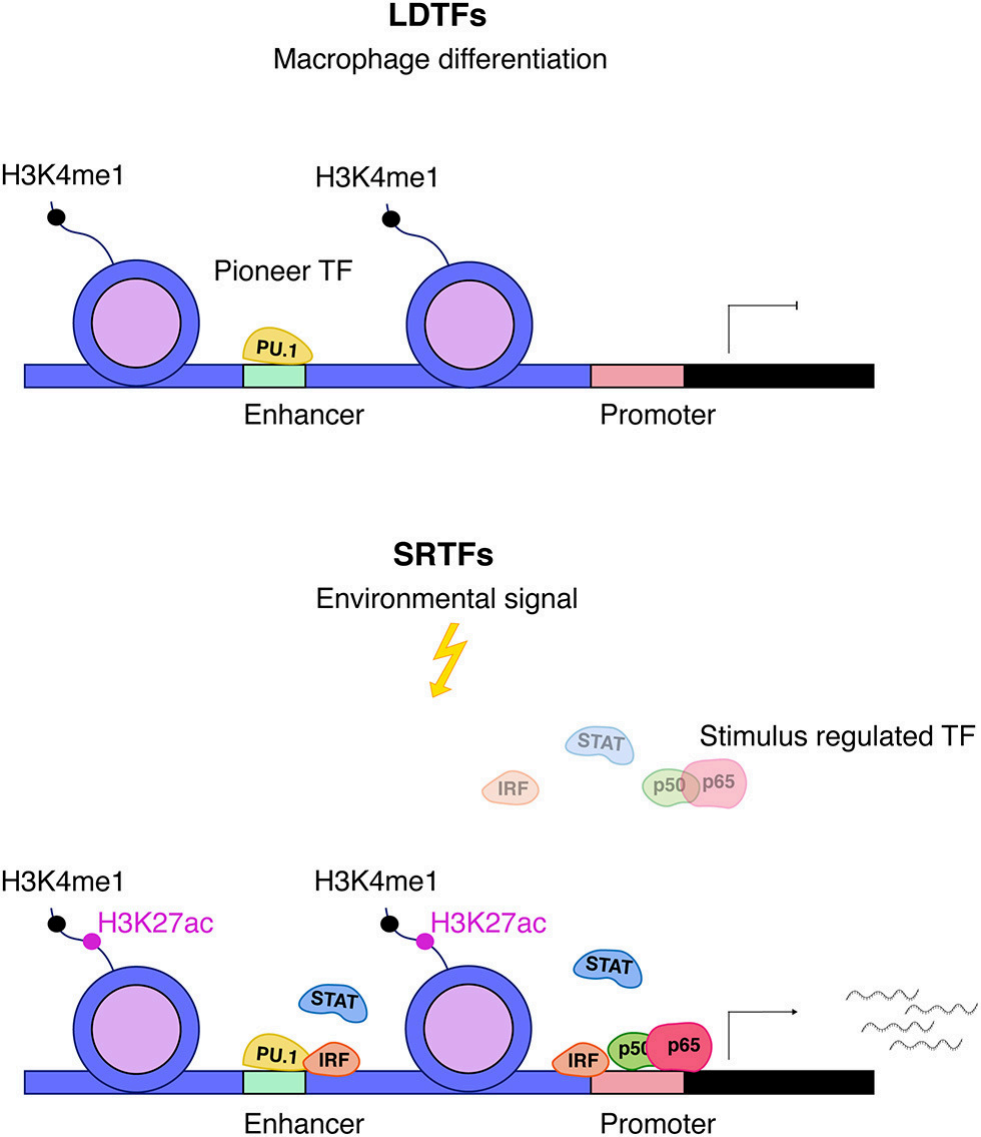
Interplay between LDTFs and SRTFs. Pu.1 is an important macrophage lineage–determining factor (LDTF) and a major driving force behind setting up macrophage enhancers for further action by SRTFs. Enhancers are distinguished by high levels of H3K4me1 and are primed by LDTFs, which further displace nucleosomes. Stimulus responsive enhancers and promoters are bound by stimulus-regulated transcription factors (SRTFs), such as STAT, NFκB, and IRF transcription factors, to direct transcriptional responses in the course of inflammation. The binding of SRTFs to primed promoters and enhancers leads to further recruitment of co-activators that deposit the activation mark H3K27ac (5).

The changes in genome structure occurring during macrophage differentiation set the stage for appropriate responses of the mature cells to challenges such as infection or inducers of sterile inflammation (Figure 2). As outlined above, this relies on the activity of LDTFs that generate poised enhancers with typical histone marks such as H3K4me1. Activation of these enhancers in differentiated cells is accompanied by the binding of a variable number of SRTFs and the deposition of additional histone modifications including the characteristic H3K27ac mark. While several LDTFs such as C/EBPα and AP1 family TFs are associated with poised enhancers in differentiating macrophages, the most prominent role in the script belongs to the Ets protein Pu.1 (59–61). Many of the Pu.1 binding sites occupied during differentiation represent EICE elements that allow for concomitant association of both Pu.1 and IRF8 [5′-GGAANNGGAAA-3′; (62)]. Thus, Pu.1 and IRF8, by specifying which of the thousands of potential enhancer sequences are accessible for transcription control, are critical in shaping a macrophage-specific chromatin landscape and the response to inflammatory stimuli. By interacting with LDTFs, IRFs, STATs, and NFκB play prominent roles in converting these enhancers into an active state that allows for contacts with the transcriptional machinery at the transcription start site (TSS). Studies such as that by Kaikkonen suggest that enhancer transcription precedes their engagement in the formation of transcription initiation complexes (61).

Not all enhancers of inflammatory gene expression are established during differentiation. Activation of terminally differentiated macrophages also causes the binding of both LDTFs and SRTFs to “latent” enhancers, i.e., sequences embedded into nucleosomes that have not been previously remodeled and marked by deposition of H3K4me1. Both IFNγ and the alternative M2 polarization factor IL4 (see below) convert latent into active enhancers by providing STAT1 and STAT6, respectively (63). Unexpectedly, the binding site in the IFNγ-activated latent enhancers is an ISRE, not a GAS sequence suggesting the participation of ISGF3 or STAT2-IRF9 complexes (29). Once activated, latent enhancers persist for some time to generate a transcriptional memory effect for subsequent stimulation. In LPS-stimulated cells composite binding sites for AP1 family and IRF8 play a prominent role in the mobilization of latent enhancers (47).

### Macrophage activation and M1 polarization

**Figure 3:**
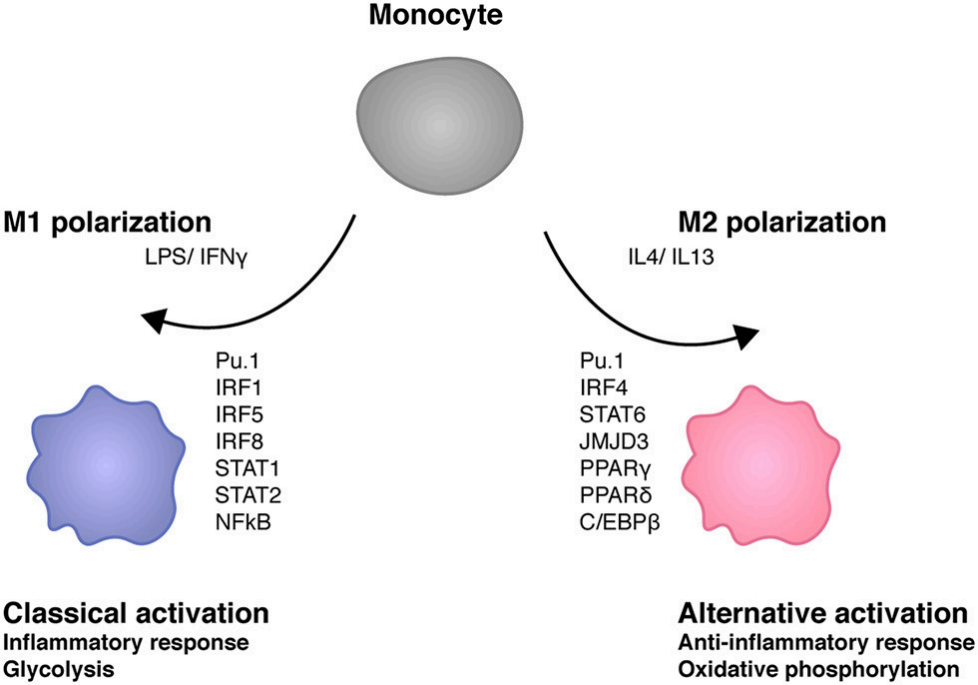
Transcription factors shaping macrophage polarity. M1 stimuli lipopolysaccharide (LPS) and interferon γ (IFNγ) trigger the activation of several transcription factors such as IRF1, IRF5, IRF8 STAT1, STAT2, and NFκB (64). M1 macrophages play key roles in inflammation as well as antibacterial responses. IL4 and IL13 induce M2 polarity in macrophages. M2 transcriptomes are determined by different transcription factors such as IRF4, STAT6, JMJD3, PPARγ, PPARδ, and C/EBPβ. M2 macrophages exert anti-inflammatory activities such as tissue repair. M1 macrophages are glycolytic whereas mitochondrial oxidative phosphorylation is required for M2 macrophage development (65).

In a simplified view macrophage activation results from signals generated by PRR such as the LPS receptor TLR4, which are amplified via JAK-STAT signal transduction by the IFNγ receptor. Polarization expresses the fact that one of several possible physiological states of a macrophage is more or less transiently established with a concomitant suppression of others (Figure 3). In case of the M1 macrophage this state is proinflammatory as well as antimicrobial and TNF/NFκB play a role in suppressing the competing M2 fate. Recent studies show that ablation of TNF responsiveness in tumor-associated macrophages (TAMs) suppresses the M1 component of their transcriptome. Further evidence for this concept comes from a report showing that the lack of TNF corresponds with an increased expression of M2 marker genes during infection with *Leishmania major* (7, 66). According to work by van den Bossche M1 polarized macrophages cannot be repolarized to M2 by IL4 treatment because the M1-typic shut-down of mitochondrial oxidative phosphorylation prevents this. The iNOS product NO plays an important role in inhibiting OxPhos, as iNOS inhibition allowed for a partial rescue of IL4-induced alternative activation in M1-polarized cells (67).

### IFNγ-independent pathways of macrophage activation and M1 polarization

The signaling network operating in IFNγ/LPS-stimulated macrophages produces several types of active SRTFs that include NFκB, IRFs, and STATs. However, many MAMPs alone produce these transcription factor activities without requiring exogenous supplies of IFNγ as follows. LPS activates NFκB both through the MyD88 and TRIF pathways downstream of its receptor TLR4. NFκB is subsequently involved in both immediate, primary, and delayed, secondary expression of proinflammatory LPS target genes (68). Primary and secondary response genes can be distinguished by a differential need for SRTF-dependent chromatin remodeling at the transcription starts (TSS) and proximal promoters (69, 70). These regions contain a high CpG content at primary response genes which impedes nucleosome formation, leaving the TSS, and promoter-proximal transcription factor binding sites accessible for initiation complex formation. In fact, many of these promoters contain paused RNA polymerases. By supporting the binding of elongation factor pTEFb to its recruitment factor, the BET protein BRD4, NFκB helps to remove the DSIF/NELF elongation block for mRNA transcription (70–73). Secondary response genes show a more delayed response, consistent with the need to restructure/modify promoter chromatin in a signal-dependent manner (68–70). Initiation complexes at these genes are established de novo. Secondary responses can be generated via regulatory feed-forward loops, i.e., the synthesis of a secondary mRNA with a transcription factor either encoded by a primary response gene, or activated by a primary response product such as TNF. An example of the former situation is the synthesis of IRF1 in the primary, STAT1-dependent response to IFNγ (52). Interactivity between NFκB and STAT/IRF pathways occurs both at primary and secondary response genes.

While TLR4 is not directly connected to a JAK-STAT pathway, the downstream TRIF pathway targets the IRF3 kinases TBK1 and IKKε, and hence the IFN-I genes (74). LPS-stimulated cells thus accumulate active STATs as a secondary response resulting from signaling by the IFN-I receptor. The same holds true for endosomal TLRs that either use the TRIF adapter for signaling (TLR3) or that form a complex containing IRF7 around the MyD88 adapter (2). In this signalosome IRF7 is phosphorylated by IKKα (75). IFN-I synthesis is also an essential outcome of signaling by all cytosolic nucleic acid receptors that signal via platforms containing the adapters MAVS or STING (3). Therefore, active NFkB, IRFs and STATs are hallmarks of pathogen-exposed macrophages even in the absence of cytokines derived from external sources such as IFNγ. The relevance of this attribute of infected or infection-exposed cells is the ability of IFN-I to provide a priming signal for resting macrophages and other cell types either resident in the surrounding tissue, or entering infected tissue in the process of inflammation (49, 76). Owing to the cooperativity between STATs and IRFs or NFκB, the deposition of ISGF3 during IFN-I priming allows for more vigorous responses to an inflammatory stimulus.

Transcription control of IFN-I genes, particularly Ifnb, is one prominent example of the interaction between different IRFs as well as between IRFs and NFκB. In macrophages the gene is constitutively bound by Pu.1/IRF8 and induction by LPS is strongly reduced in cells lacking functional IRF8 (47). LPS or viral infection stimulate the promoter binding of IRF3 and/or IRF7 as well as NFκB and the subsequent recruitment of chromatin remodeling and modifying enzymes (77). The analysis of the active Ifnb promoter culminated in an atomic model of its enhanceosome (78). It contains not only IRFs3/7 and NFκB, but also the AP1 family members ATF2 and c-Jun.

The cooperation between NFκB and IRF3 shows additional levels of complexity in TLR responses of macrophages. Two studies are consistent with the idea that the two transcription factors can act as coactivators for each other. On the one hand, NFκB function at a subset of its binding sites requires direct interaction with IRF3 (53). On the other hand, p65 is tethered to ISRE subsets during macrophage TLR4 or TLR9 signaling. In LPS-treated macrophages around 100 ISREs bound these complexes and the corresponding genes were selectively inhibited by agonists of the glucocorticoid receptor [GR; (54)]. The data further demonstrate sensitivity of the interaction between IRF3 and the RelA RHD to disruption by the GR *in vitro*. The two studies reveal that some inflammatory genes are controlled by NFκB/IRF interaction without having promoter binding sites for both. Genome-wide DNA binding data in virus-infected cells further confirmed the impact of gene co-regulation by IRF3 and NFκB (79). An alternative experimental approach supports the importance of this finding by using a virus mutant with reduced ability to suppress NFκB activation. In IRF7-deficient cells the increased NFκB activation compared to wt virus partially rescued inflammatory gene expression and antiviral immunity (80), suggesting that a higher dose of NFκB activity compensates for the loss of IRF7 at coregulated genes.

Similar to IRF3, IRF5 is recruited to inflammatory genes and is essential for their efficient transcription. In LPS-treated macrophages NFκB assists IRF5 in binding to DNA, and the two factors set up a unique “inflammatory” IRF5-RelA cistrome which is best explained by the presence of consensus NF-κB and a composite Pu.1-ISRE element (81). Conditional deletion of IRF5 in macrophages is incompatible with M1 polarization (82).

IFN-I production in response to inflammatory stimuli produces transcriptional activity of transcription factor ISGF3, the STAT1/STAT2/IRF9 heterotrimer. ISGF3 exemplifies a direct physical STAT/IRF contact (37). According to the JAK-STAT paradigm described above, ISGF3 is the terminal component of IFN-I and IFN-III signaling. Whereas, a large fraction of IFNγ-inducible genes essentially depends on multimerized STAT1 dimers (83), there is currently no evidence that STAT1 dimers make important contributions to IFN-I or IFN-III signaling. As will be discussed in more detail below, genes regulated by STAT1 dimers are characterized by frequent cooperativity with IRF1, IRF8 and, at least for some genes, IRF7 (48, 52, 84–86). Conventional ISGs, i.e. ISGF3-dependent genes responding with a strong transcriptional increase to IFN-I do not require IRF1 (87), but a large fraction shows ISGF3 binding at or near Pu.1 and IRF8 (47, 62). Correspondingly, about 20% of total ISGs show diminished responses to IFNβ in cells expressing the IRF8 R294C mutant which is unable to interact with Ets family proteins and thus strongly impaired in its ability to bind DNA at composite binding sites (88). The ISREs of such ISGs contain the expected 5′ GGAA motif that allows for simultaneous association of IRF8 and Pu.1. Notwithstanding the neighborhood of ISGF3 and IRF8 at macrophage promoters, activity of the ISGF3 complex shows greater independence from ancillary IRFs than STAT1 dimers. One reason for this is the potent transactivating domain (TAD) of STAT2 compared with that of STAT1 (89). In fact, expression of a fusion protein of IRF9 with the STAT2 TAD largely recapitulates the transcriptional response to IFN-I (90).

In non-hematopoietic cells ISGs require a signal-independent nucleosome rearrangement prior to ISGF3 binding. It is executed by the mammalian SWI/SNF (or BAF) remodeling complex including the ATPase BRG1 (91). In addition, recent work has shown that ISGF3 binding causes additional IFN-dependent remodeling, shown by the appearance of open chromatin regions in ATAC-Seq or MNase I sensitivity experiments (92, 93). Histone exchange also takes place and results in the removal of the repressive variant H2A.Z. It will be interesting to determine how these non-hematopoietic remodeling events compare to chromatin opening during macrophage differentiation.

**Figure 4:**
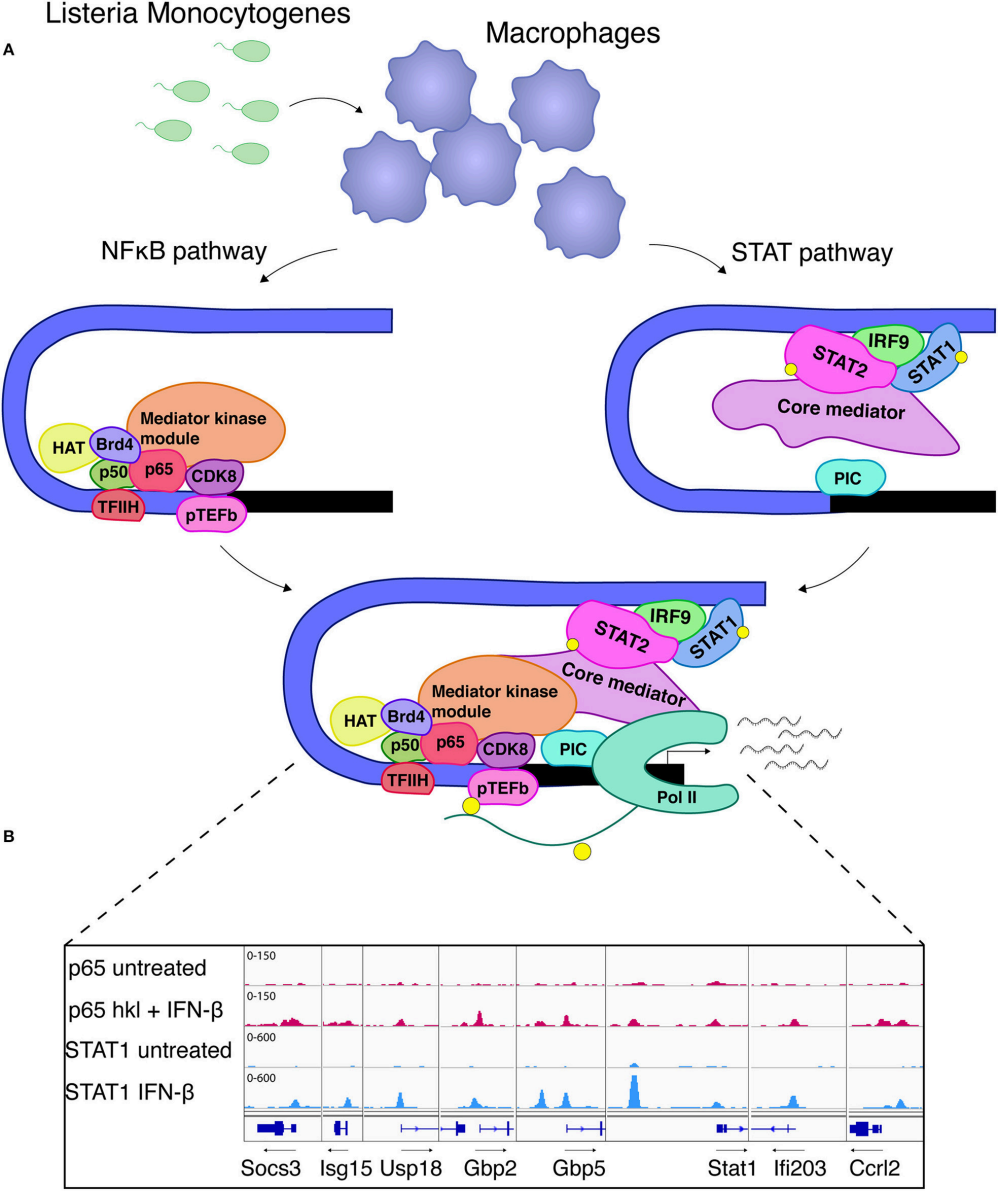
STAT-NFκB cooperativity shapes the transcriptional response to *Listeria monocytogenes*. **(A)** NFκB association with the Nos2 promoter is the initial step and leads to recruitment of TFIIH which is further required for Pol II phosphorylation. Brd4 stabilizes the NFκB-TFIIH complex. In addition, association of HAT and the mediator kinase module (CDK8) strongly depend on NFκB. ISGF3, which recruits the core mediator, is essential for the formation of a pre-initiation complex (PIC) and further provides a critical prerequisite for TFIID and RNA polymerase II (Pol II) binding (49–51). These references also describe the experimental procedures for separate analysis of STAT and NFκB signaling during *L. monocytogenes* infection. **(B)** Binding of NFκB RelA/p65 and STAT1 to promoters of co-regulated ISGs. The igv browser tracks show induced RelA/p65 binding in macrophages treated with IFNβ and heat-killed Listeria (hkl). STAT1 binding sites are shown from IFNβ-stimulated macrophages. A ChIP-seq data set described in reference 50 was used.

Despite the largely self-sufficient mode of ISGF3 action at many conventional ISGs, some genes expressed in MAMP-exposed or infected macrophages demonstrate a different behavior. These genes are referred to as unconventional ISGs (76) or synergy genes (50) because they respond poorly to IFN-I alone, instead requiring an additional PRR-derived signal. In many cases NFκB is essential to provide this second signal (50). By conservative estimate around 130 unconventional ISGs show synergistic transcriptional responses owing to ISGF3 and NFκB occupancy in macrophages infected with the intracellular bacterial pathogen *Listeria monocytogenes*. They include the genes encoding iNOS (NOS2) or IL6. Mechanistically the cooperativity at these two genes is explained by NFκB predominance in the recruitment of histone-modifying enzymes, the BET protein Brd4 and the TFIIH and pTEFb complexes needed for the phosphorylation of the RNA polymerase II (Pol II) carboxyterminal domain (CTD; Figure 4A). In contrast, ISGF3 plays a dominant role in the promoter recruitment of the general transcription factor TFIID and the formation of a complete initiation complex including Pol II. Both transcription factors cooperate in the recruitment of the mediator complex with core and kinase modules (49–51). Whether these mechanisms apply to all or a majority of genes showing NFκB/ISGF3 cooperativity remains to be determined. We have found that some of the conventional ISGs such as Isg15, Gbp, or Stat1 also have binding sites for NFκB and these sites are occupied during infection with *L. monocytogenes* [Figure 4B, data set as in (50)]. This suggests an even larger input of NFκB signaling into transcriptional responses to IFN. From the immunological standpoint the cooperative activation mode is useful because it allows to better dose signal strength at promoter level. Furthermore, NFκB can generate a state of transcriptional short-term memory for ISGF3, i.e., providing an NFκB-inducing signal alone generates a cooperative response even when the IFN signal follows up to 24 h later (49). This effect is similar to the latent enhancer activation described above, or to the priming effect of IFNγ described below. During an inflammatory response the kinetics of exposure to the many environmental signals differ for individual cells, thus these memory mechanisms appear highly advantageous. In support of this notion a study by Park et al. confirms the ability of both TNF and IFNα to reprogram the human macrophage epigenome, thus altering inflammatory responses to TLR4 stimulation. Preexposure with TNF may tolerize genes with NFκB sites or lead to synergism with LPS in genes with ISRE/IRF and AP1 elements. IFN pretreatment can counteract the tolerizing effects of TNF (76).

### The transcriptional response to IFNγ–its contribution to macrophage activation and inflammation

IFNγ is the macrophage-activating cytokine produced in the course of an inflammatory responses by innate lymphocytes such as NK or ILC1 cells, but also via an innate response of T lymphocytes. Immunological effects of IFNγ are to a large extent established *de novo* and require extensive chromatin landscape changes (94). Formation of STAT1 dimers (or GAF) by the IFNγ receptor complex is sufficient for the transcription of a relatively small number of primary response genes with GAS promoter elements. Among these are the Irf1 and Irf8 genes. IRF1 and IRF8 production is necessary for a delayed transcriptional of many secondary IFNγ-induced genes, represented by e.g., the GBP family, the gp91Phox, Nos2 (iNOS), or the Ciita gene encoding the master regulator of MHC II expression (48, 52, 84, 85, 95–97). In addition, our recent data in IFNγ-treated macrophages reveal a surprisingly large contribution of ISGF3 to the IFNγ-induced transcriptome (29).

**Figure 5:**
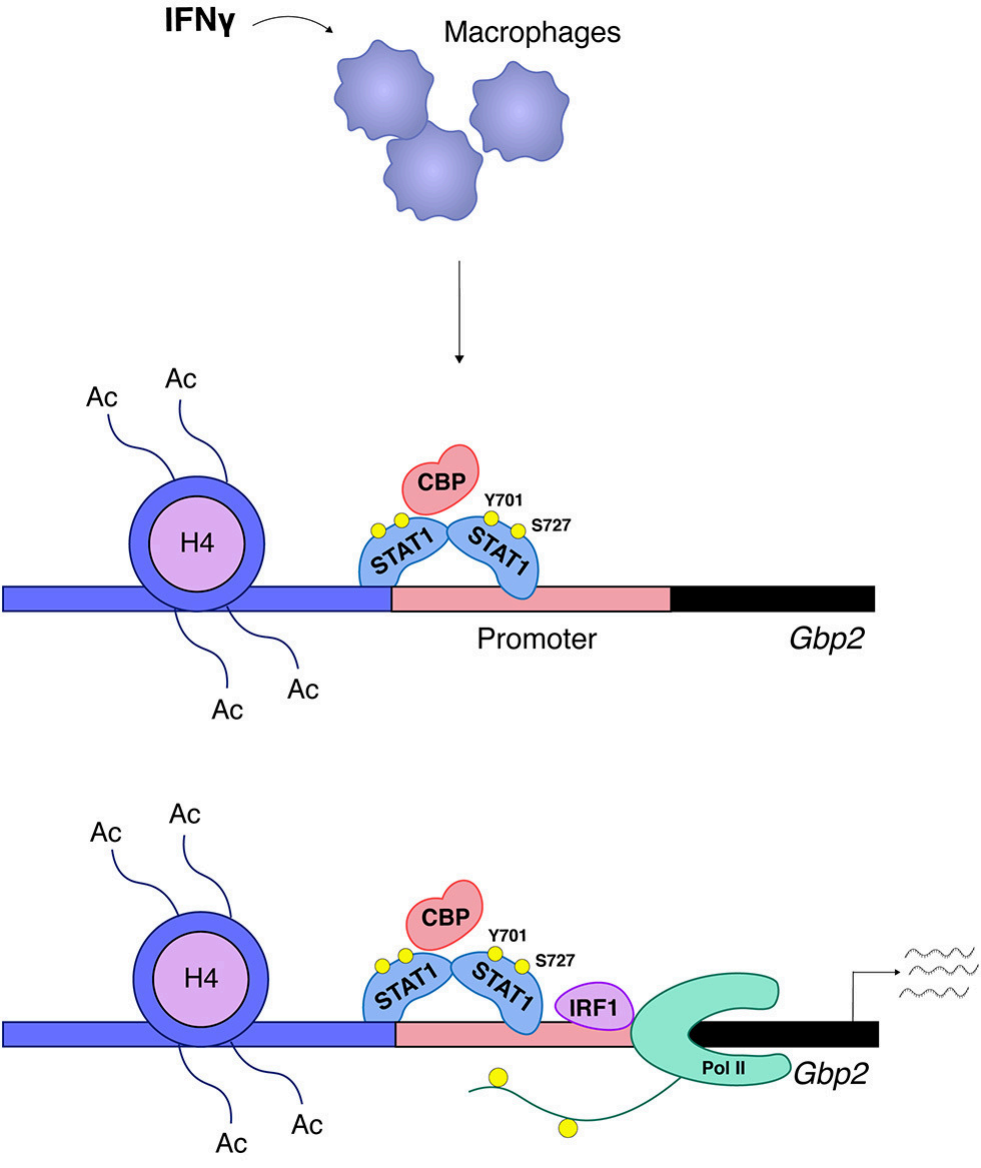
STAT1 and IRF1 synergistically drive expression of Gbp2. A large group of IFNγ-induced genes such as Gbp2 requires both STAT1 and IRF1 for transcriptional activation (52). STAT1 associates with the Gbp2 promoter and is responsible for the ordered recruitment of the coactivator/histone acetyl transferase CREB-binding protein (CBP) and histone hyperacetylation. CBP recruitment requires phosphorylation of the STAT1 TAD at S727. Irf1 is a STAT1 target gene and, following IRF1 synthesis, its association with the Gbp2 promoter follows that of STAT1, but in respective knockout cells the two transcription factors bind without requiring each other's presence. RNA polymerase II (Pol II) association with the Gbp2 promoter requires both STAT1 and IRF1, but only IRF1 is found in a complex with RNA polymerase II.

Increased amounts of IRF8 in LPS or IFNγ-treated cells allow the transcription factor to occupy landing sites in addition to those established during macrophage differentiation. This is important because similar to ISGs, many IFNγ-inducible promoters are prebound with Pu.1 and a subfraction of these are associated with Pu.1/IRF8. In this situation Pu.1/IRF8 binding occurs via EICE sequences, whereas inducible binding of IRF8 occurs via ISRE, but not via GAS sites (47). IRF1 appears to have a minor-if any- role in enhancer establishment during differentiation, but its role in signal-dependent gene induction is essential and not redundant with other IRFs. Based on their IRF requirement, a recent report by Langlais and colleagues distinguishes two types of IFNγ-induced gene clusters. The first is characterized by prebound Pu.1/IRF8 and its ISRE sequence will associate with both IRF8 and IRF1 in IFNγ-treated cells. The second type of IFNγ-induced genes is prebound by Pu.1 alone and its ISRE will associate with IRF1 only. Both clusters show a large overlap with STAT1 binding (48). Thus, STAT1 in the IFNγ response acts both as an inducer of the primary response genes IRF1 and IRF8 and then cooperates with these IRFs in the second tier of the transcriptional response. There is a very limited number of studies providing a mechanistic explanation why IRFs and STATs together potently induce transcription where either of them alone fails to do so. Two studies correlated the presence of IRF8 with the establishment of constitutive H3K27 acetylation (47, 48) and one of these links IRF1 binding with the IFNγ-induced increase of this mark (48). At genes with EICE sequences IRF8 participates in the recruitment of STAT1, leaving it open which of the two is the histone acetyl transferase (HAT)-recruiting factor (47). In non-hematopoietic cells our data suggest a crucial role of STAT1 in the recruitment of the HAT CBP/P300 to the promoter of the IFNγ-inducible GBP2 gene with no or very little contribution of IRF1. CBP recruitment required phosphorylation of the C-terminal S727 in the STAT1 TAD. The two transcription factors displayed no interdependence of DNA binding, but were equally needed for cooperative recruitment of RNA Pol II [(52), Figure 5]. With the discovery of different clusters of IFNγ-induced genes in macrophages, regulatory heterogeneity may apply to non-hematopoietic cells as well and the data with the GBP promoter are likely to represent only a subfraction of IFNγ-induced genes. Studies by El Hassan et al. in epithelial cells show that IRF1 occupies a large number of sites without STAT1, but, conversely, STAT1 is mostly co-associated with IRF1 [(97), this appears to be different in macrophages (48)]. The larger number of IRF1 binding sites may be explained by a role of IRF1-binding enhancers in the formation of a 3D promoter structure as reported by the same group for the gene encoding the MHC II master regulator CIITA (96).

We have briefly mentioned the participation of IFNγ/STAT1 in the mobilization of latent enhancers in murine macrophages. Consistent with this Qiao et al. report studies in human macrophages showing that IFNγ primes LPS-responsive genes with STAT/IRF binding sites without necessarily activating their transcription (98). Examples are the genes encoding IL6 and IL12. IFNγ priming leads to a massive increase in their subsequent LPS responsiveness. Whether this is mechanistically related to the priming of the Il6 and Nos2 (iNOS) genes by type I IFN remains to be determined. However, the data suggest that cooperativity between NFκB and STATs may contribute to the increased inflammatory response after priming with IFNγ from exogenous sources much as it does in case of proinflammatory stimuli and endogenously produced IFN-I.

### M2 polarization-the cross-repression of M1 and M2 genes

Macrophages undergo “alternative” activation and M2 polarization when exposed to the type II immunity cytokines IL4 and IL13. They lack proinflammatory gene expression and have strongly reduced antimicrobial effector functions. Instead, they typically express genes allowing for tissue repair and fibrosis. M2 type genes such as Arginase1 (Arg1) or the mannose receptor Mrc1 are expressed in adipose tissue macrophages, but also in tumor-associated macrophages or in macrophages fighting parasitic infections (8, 64). Unlike M1 polarization, M2-polarized macrophages can be converted to an M1 type *in vitro* by treatment with IFNγ/LPS (67).

The apical transcription factor specifying the M2 state is STAT6, activated by the IL4 and IL13 receptors. Many of the typical M2 genes are direct targets of STAT6. IL4 also induces an IRF family member, IRF4. The human Irf4 gene contains a GAS element, suggesting it is under direct control of STAT6 (99, 100). Transcriptional induction of the Irf4 gene also requires the histone demethylase JMJD3 to remove the repressive H3K27me3 histone mark in the vicinity of the Irf4 transcription start. This demethylation step is of critical importance as Jmjd3^−/−^ macrophages do not undergo M2 polarization during helminth infection (101). More recent knockdown studies in human monocytes suggest an enhancement of JMJD3 expression by STAT6 (100). This suggests that STAT6 is apical to both JMJD3 and IRF4. In specifying M2 transcriptomes STAT6 and IRF4 interact with other transcription factors, particularly PPARγ, C/EBPβ, and KLF4. Gene deficiencies for these transcription factors reduce the M2 potential, as do those for STAT6 and IRF4 (8). Different combinations of these transcription factors may specify distinct M2ish transcriptomes in animals (64). For example, PPARγ is instrumental in arranging the lipid metabolism of adipose tissue macrophages.

STAT6 is unique among STATs by preferentially associating with GAS containing 4 spacer nucleotides between half sites (5′-TTCN_4_GAA-3′). This explains in part why its target genes are different from those activated by STAT1. On the other hand, genome-wide analysis of STAT1 and STAT6 binding to GAS elements revealed a large number of sites occupied by STAT1 and STAT6, respectively, in IFNγ or IL4-stimulated cells (102). Thus, epigenetic mechanisms and/or the cooperation with other M2 transcription factors are likely to further contribute to the distinction between STAT6 and STAT1-induced transcriptome changes. Although IRF4 is most likely a primary STAT6 target in analogy to the situation with STAT1 and IRF1/IRF8, we are not aware of a similarly important interaction of STAT6 and IRF4 at the level of common target promoters. In a recent study, an important function is assigned to STAT6 in cross-repressing M1 genes. In IL4-treated macrophages STAT6 represses genes at steady state and, in addition, renders proinflammatory genes unresponsive to a subsequent LPS challenge. Suppression requires HDAC3 activity and causes decreased promoter binding of LDTFs (e.g., Pu.1, C/EBP) as well as the p300 histone acetyl transferase. More than 600 LPS-induced genes show an overlap of NFκB and STAT6 binding sites (GAS), 70% of which are inhibited by IL4 and in 11.5% of them IL4 inhibited RelA/p65 binding (57). These genome-wide data are consistent with a model of competitive enhancer association as one of several mechanisms by which STAT6 represses NFκB activity. This model agrees with an earlier study of the E- selectin promoter suggesting direct competition of STAT6 and NFκB at overlapping binding sites (56). IRF4 stimulates gene expression via ISRE elements. It forms ternary complexes with Pu.1 and IRF8 on composite ISREs. However, the data are conflicting with regard to the outcome of complex formation on ISG transcription with some suggesting an inhibitory activity and repression of IRF1 activity (103, 104) and others supporting a stimulatory role (105).

Just as M1 genes are suppressed in M2 macrophages, the inverse situation is established in M1 polarized cells. In human macrophages transcription factor MAF regulates the expression of a subset of M2 genes. Fifteen percent of the genes repressed by IFNγ treatment are MAF targets. MAFb is a bZip transcription factor important for the development of macrophage-dendritic cell progenitors to monocytes (106). The work of Kang et al. demonstrates that IFNγ treatment causes a disassembly of MAF-associating enhancer sequences. Accompanying changes were a loss of enhancer transcription, of LDTF binding (Pu.1, CEBPβ), cohesin association and of the H3K27ac mark as a beacon of transcriptionally active genes. At the same time IFNγ deposits LDTFs at latent enhancers. Disassembled MAF enhancers also lose accessibility, determined by the disappearance of ATACseq signals. Chromatin closing at MAF enhancers contrasts with the majority of IFNγ-repressed genes that appear to be subject to a different inhibitory mechanism. A complex enhancer regulating MAF expression loses activating histone marks upon IFNγ treatment, consistent with decreased MAF levels in such cells. MAF family members are known to interact with Ets family proteins, suggesting that M2 enhancer accessibility may be regulated via Pu.1/MAF interaction. Another report by Ivashkiv's group showed that in human macrophages a small number of genes is stably repressed by IFNγ via H3K27me3 deposition by the PRC2 complex including the histone methylase EZH2 (107). These elegant studies provide important insight how IFNγ tips the polarity balance by repressing M2 genes. The studies do not address how the repressive mechanisms are linked to signals from the IFNγ receptor or the STAT/IRF network it activates. This will be an important task for future work. Likewise, the similarities and differences to mouse macrophages will interesting to decipher.

In a different approach, Piccolo and colleagues addressed the question how mouse macrophages are reprogrammed by a simultaneous encounter with the opposing IFNγ and IL4 (102). The authors report that each cytokine cross-inhibits genes of the opposite pole, but the impact of IFNγ dominates, most likely because it exerts a global effect on the binding of STAT6. In contrast, the repressive effect of IL4 on IFNγ-induced genes is more selective. Promoters containing GAS and ISRE elements only are spared from repression whereas more complex promoters that include sites for AP1 transcription factors with JunB subunits, such as the Nos2 gene, are repressed. In line with the study in human macrophages (108), MAFb-regulated genes are also enriched among IFNγ-repressed genes. The authors speculate that the inability of IL4 to globally suppress all IFNγ activity allows macrophages to maintain essential immunological functions such as the antiviral state. However, an immunological assessment of IL4/IFNγ co-treated macrophages has not been carried out. Interestingly, combining the finding that STAT6 and STAT1 both bind many GAS elements with the observed suppression of STAT6 binding by IFNγ lends further support to the direct competition model as one mechanism explaining the cross inhibition of polarization genes.

### Sequels of the inflammatory response: LPS tolerance and macrophage training

Chronic stimulation of macrophages with LPS causes a state of tolerance in which many inflammatory genes are refractory to further stimulation with LPS. The mechanisms contributing to this refractory state are manifold and many are not at promoter level, but rather affect TLR signaling and the activation of inflammatory transcription factors (109). A notable exception is the NFκB pathway. NFκB binding sites are enriched in tolerized genes (110), supporting an earlier notion that NFκB1/p50 forms repressive dimers on such sites (111). Macrophages lacking NFκB1 cannot be tolerized by LPS (112). Yan et al further demonstrate that p50 recruits HDAC complexes to tolerized genes. In human monocyte-derived macrophages LPS pretreatment leads to a tolerant state with extensive changes in histone modifications (enhancer marks) in which a fraction of LPS-induced genes shows an absent or reduced response to a second LPS stimulus. IRF (particularly IRF8) and STAT motifs are strongly enriched in genes showing partial unresponsiveness. IRF1, IRF8, STAT2, and STAT5 themselves are among highly tolerized genes. Interestingly, tolerization can to a large extent be reversed by the yeast MAMP β-glucan. This substance is associated with “trained immunity” (113). IFNγ pretreatment reverses effects of the tolerized state by maintaining chromatin remodeling and accessibility at LPS-induced genes (114). A seminal study on the topic showed that chromatin modifications and accessibility acquired during tolerization distinguish between proinflammatory cytokine genes and antimicrobial genes. While the former show a loss of remodeling and activating histone marks, these are retained on the latter. Consistently, the proinflammatory group enters a state of repression while the latter maintains inducibility (115). This suggests that in the tolerant state inflammation is dampened whereas antimicrobial defense remains. However, LPS tolerance is also thought to underlie the immunoparalysis of post-septic patients.

### Trained immunity

Trained immunity denotes a state of innate immunological memory resulting in macrophages and other innate cells from the previous encounter of an inflammatory and activating stimulus (116). LPS tolerance is one form of trained immunity. Training resulting from microbes such as BCG or *Candida albicans* or the yeast-associated molecular pattern β-glucan enhances subsequent responses of the innate immune system to different microbial infections. The trained state is accompanied by persistent metabolic and epigenetic changes. Epigenomic alterations show partial overlap, but are clearly distinct between LPS-tolerized and β-glucan-trained human macrophages (117). At the level of transcription training is explained by persistence of activating chromatin marks and regulatory site accessibility, particularly at previously latent enhancers. The causes for these genome changes are not understood. One study suggests an IFNγ-dependent role for STAT1 in training by *C. albicans*, but not β-glucan (118).

## STATs and IRFs in dendritic cell (DC) development and function

For a comprehensive review of transcription factors in the development of DC subsets the reader is referred to the following reviews (119, 120).

### DC development

Both STATs and IRFs play important roles in the generation of different DC lineages and both influence each other's activities. STAT3, downstream of the Flt3 receptor, is required for the generation of most likely all DC lineages *in vitro* and has a clear role in plasmacytoid DC (pDC) development *in vivo* (121). Constitutive IRF7 expression is thought to accompany the development of pDC precursors into IFN-I-producing cells (IPC), i.e., cells with the ability to rapidly synthesize large quantities of IFN-I in response to TLR ligands (122). Recent studies suggest that pDC precursors diversify in response to a single TLR ligand and not all the resulting subpopulations are IFN-I-producing cells (123). Single cell RNA sequencing of human pDC suggests that only a small subpopulation is stimulated to produce IFN-I. IFN-I production of this population results from stochastic events rather than from developmental predetermination and IRF7 is indeed not a prognostic marker for future IFN-I production (124). Based on this study the concept how IFN-producing cells arise may have to be revised.

STAT5 is required for the production of myeloid DC by GM-CSF *in vitro*. It also mediates the suppressive function of GM-CSF on pDC development (58). Type I IFN stimulate DC maturation (125) and were shown in a recent study to regulate the glycolytic switch required for DC activation (126). In contrast, IFN-I suppress CD8^+^ DC generation *in vitro* and during viral infection *in vivo*. Intriguingly, this effect was shown to require STAT2, but not STAT1 (127). The seeding of Payer's patches with pDC also requires IFN-I/STAT activity (128).

As during macrophage polarization, IRF8 and IRF4 determine different DC fates. IRF8 is critical for the development of pDC, tissue-resident CD103^+^ DC, CD8^+^ DC and Langerhans cells (129–133). Intriguingly, the R294C mutant allows to distinguish the role of IRF8 in the generation of pDC vs. CD8^+^ and CD103^+^ DC with the former being unaffected and development of the latter being inhibited (88, 131, 134). The property of this mutation to disrupt the interaction between IRF8 and Pu.1 (88) supports the assumption that composite Pu.1/IRF8 elements are important for CD103^+^ and CD8^+^ DC, but not for pDC. The IRF8 transcriptional network in CD103^+^ and CD8^+^ DC includes Id2, BATF3, and Notch2, whereas in pDC IRF8 functionally interacts with E2-2 and Bcl-11a (119). The suppressive effect of GM-CSF on pDC development requires STAT5 binding to a GAS in the IRF8 promoter where it inhibits IFN/STAT1-mediated upregulation of IRF8 expression (58).

IRF4 is expressed in cDC expressing CD4 and CD11b (also called cDC2) in a transcriptional network including RelB and Notch2 (119, 135). IRF4 may support cDC2 function more than their development. cDC2 are linked to both type 2 and type 3 immunity (135).

### DC activation

Similar to the studies defining the hierarchical action of transcription factors and the distinction between LDTFs and SRTFs in macrophages, Garber and colleagues defined analogous networks of preexisting pioneers (LDTFs) for lineage commitment, broad binders for priming, and dynamic factors (SRTFs) for execution (136). The authors used myeloid, IRF4^+^ DC, generated *in vitro* with GM-CSF, for stimulation with LPS. Prominent LDTFs were Pu.1 and C/EBP as in macrophages. However, whether they occupy different sites in the two cell types has not been determined. In the LPS response the authors distinguished immediate and delayed response gene clusters that differed in priming factors (immediate: IRF4, JUNB, ATF3, EGR2, MAFf; delayed: IRF4, JUNB, ATF3) and dynamic association of the SRTFs IRF1 and NFκB. As might be expected from the kinetics of IFN production during the LPS response, STATs 1 and 2 associated with the delayed, but not the immediate LPS-responsive gene cluster.

The data in DC support the notion that the molecular principles governing enhancer accessibility, priming, and signal-regulated response are very similar between macrophages and DC, although the detailed usage of transcription factors for each stage of the transcriptional response during inflammation may differ.

## Concluding remarks

This review is focused on the role of macrophages in inflammation, with lesser attention to DC or non-hematopoietic cells. We realize this is only one chapter of a complex story but, based on available data, have focused on the cells allowing for the closest inspection of transcriptional networks in inflammation. Detailed views of the inflammatory response in other cells of the innate immune system are needed. In an organismic context, macrophages are shaped by their local environment (137). The impact of the resulting diversity on inflammatory responses remains to be determined. In future work ongoing large-scale efforts will integrate dynamic changes of the 3D genome structure into current knowledge about regulatory networks (138) and cell atlases based on single cell transcriptomes will further resolve distinct cell populations of the inflammatory response (139). However, while genome-wide perspectives have yielded new insight at an amazing pace in recent years and will continue to do so, future research needs to follow up on these data with biochemical approaches toward mechanisms of transcription factor cooperativity and antagonism. For example, it will be exciting to determine the different modes of action of LDTFs and SRTFs or to provide further insight how combinations of SRTFs feed into a common overall mechanism of promoter activation. Determining dynamic structures of the complexes they form at near atomic resolution will be invaluable support for such efforts (140). Cas9-mediated editing is and will be an extremely useful tool to test the impact of regulatory DNA (141). The recent past has given us a panoply of powerful new tools to advance our understanding of transcriptional mechanisms behind the inflammatory response.

## Author contributions

All authors listed have made a substantial, direct and intellectual contribution to the work, and approved it for publication.

### Conflict of interest statement

The handling Editor declared a past collaboration with one of the authors TD. The remaining author declares that the research was conducted in the absence of any commercial or financial relationships that could be construed as a potential conflict of interest.

## References

[1] Medzhitov R. Inflammation 2010: new adventures of an old flame. Cell (2010) 140:771–6. 10.1016/j.cell.2010.03.006 20303867

[2] O'Neill LAJ. When signaling pathways collide: positive and negative regulation of toll-like receptor signal transduction. Immunity (2008) 29:12–20. 10.1016/j.immuni.2008.06.004 18631453

[3] Hu M-M Shu H-B. Cytoplasmic mechanisms of recognition and defense of microbial nucleic acids. Annu Rev Cell Dev Biol. (2018) 34: 357–9. 10.1146/annurev-cellbio-100617-062903 30095291

[4] Natoli G Ghisletti S Barozzi I. The genomic landscapes of inflammation. Genes Dev. (2011) 25:101–6. 10.1101/gad.2018811 21245163 PMC3022255

[5] Glass CK Natoli G. Molecular control of activation and priming in macrophages. Nature Immunol. (2016) 17:26–33. 10.1038/ni.3306 26681459 PMC4795476

[6] Martinez FO Gordon S. The M1 and M2 paradigm of macrophage activation: time for reassessment. F1000Prime Rep. (2014) 6:13. 10.12703/P6-13 24669294 PMC3944738

[7] Ostuni R Kratochvill F Murray PJ Natoli G. Macrophages and cancer: from mechanisms to therapeutic implications. Trends Immunol. (2015) 36:229–39. 10.1016/j.it.2015.02.004 25770924

[8] Murray PJ. Macrophage polarization. Annu Rev Physiol. (2017) 79:541–66. 10.1146/annurev-physiol-022516-034339 27813830

[9] Chávez-Galán L Olleros ML Vesin D Garcia I. Much more than M1 and M2 macrophages, there are also CD169(+) and TCR(+) macrophages. Front Immunol. (2015) 6:263. 10.3389/fimmu.2015.00263 26074923 PMC4443739

[10] Levy DE Darnell JEJ. Stats: transcriptional control and biological impact. Nat Rev Mol Cell Biol. (2002) 3:651–2. 10.1038/nrm909 12209125

[11] Stark GR Darnell JE. The JAK-STAT pathway at twenty. Immunity (2012) 36:503–14. 10.1016/j.immuni.2012.03.013 22520844 PMC3909993

[12] Hayden MS Ghosh S. NF-κB, the first quarter-century: remarkable progress and outstanding questions. Genes Dev. (2012) 26, 203–14. 10.1101/gad.183434.111 22302935 PMC3278889

[13] Oeckinghaus A Hayden MS Ghosh S. Crosstalk in NF-κB signaling pathways. Nature Immunol. (2011) 12:695–708. 10.1038/ni.2065 21772278

[14] Tamura T Yanai H Savitsky D Taniguchi T. The IRF family transcription factors in immunity and oncogenesis. Ann Rev Immunol. (2008) 26:535–84. 10.1146/annurev.immunol.26.021607.090400 18303999

[15] Negishi H Taniguchi T Yanai H. The Interferon (IFN) class of cytokines and the IFN regulatory factor (IRF) transcription factor family. Cold Spring Harb Perspect Biol. (2017). 10.1101/cshperspect.a028423. [Epub ahead of print]. 28963109 PMC6211389

[16] Mertens C Zhong M Krishnaraj R Zou W Chen X Darnell JE. Dephosphorylation of phosphotyrosine on STAT1 dimers requires extensive spatial reorientation of the monomers facilitated by the N-terminal domain. Genes Dev. (2006) 20:3372–81. 10.1101/gad.1485406 17182865 PMC1698445

[17] Decker T Kovarik P Meinke A. Gas elements: a few nucleotides with a major impact on cytokine-induced gene expression. J Interferon Cytokine Res. (1997) 17:121–34. 9085936 10.1089/jir.1997.17.121

[18] Honda K Taniguchi T. IRFs: master regulators of signalling by Toll-like receptors and cytosolic pattern-recognition receptors. Nat Rev. (2006) 6:644–58. 10.1038/nri1900 16932750

[19] Escalante CR Yie J Thanos D Aggarwal AK. Structure of IRF-1 with bound DNA reveals determinants of interferon regulation. Nature (1998) 391:103–06. 10.1038/34224 9422515

[20] Tanaka N Kawakami T Taniguchi T. Recognition DNA sequences of interferon regulatory factor 1 (IRF-1) and IRF-2, regulators of cell growth and the interferon system. Mol Cell Biol. (1993) 13:4531–8. 7687740 10.1128/mcb.13.8.4531-4538.1993 PMC360068

[21] Mamane Y Heylbroeck C Genin P Algarte M Servant MJ LePage C. Interferon regulatory factors: the next generation. Gene (1999) 237:1–14. 10524230 10.1016/s0378-1119(99)00262-0

[22] Taniguchi T Ogasawara K Takaoka A Tanaka N. Irf family of transcription factors as regulators of host defense. Ann Rev Immunol. (2001) 19:623–55. 10.1146/annurev.immunol.19.1.623 11244049

[23] Chen X Vinkemeier U Zhao Y Jeruzalmi D Darnell JEJ Kuriyan J. Crystal structure of a tyrosine phosphorylated STAT-1 dimer bound to DNA. Cell (1998) 93:827–39. 9630226 10.1016/s0092-8674(00)81443-9

[24] Schmitz ML Baeuerle PA. The p65 subunit is responsible for the strong transcription activating potential of NF-kappa B. EMBO J. (1991) 10:3805–17. 10.1002/j.1460-2075.1991.tb04950.x 1935902 PMC453117

[25] Ghosh G Wang VY-F Huang D-B Fusco A. NF-κB regulation: lessons from structures. Immunol Rev. (2012) 246:36–58. 10.1111/j.1600-065X.2012.01097.x 22435546 PMC4543363

[26] Reich NC Liu L. Tracking STAT nuclear traffic. Nat Rev Immunol. (2006) 6:602–12. 10.1038/nri1885 16868551

[27] Levy DE Kessler DS Pine R Reich N Darnell JEJ. Interferon-induced nuclear factors that bind a shared promoter element correlate with positive and negative transcriptional control. Genes Dev. (1988) 2:383–93. 3371658 10.1101/gad.2.4.383

[28] Mariani M Dasmeh P Fortin A Kalamujic M Caron E Harrison AN RNASeq analysis identifies non-canonical role of STAT2 and IRF9 in the regulation of a STAT1-independent antiviral and immunoregulatory transcriptional program induced by IFNβ and TNFα. bioRxiv [preprint] (2018). 10.1101/273623

[29] Platanitis E Demiroz D Capelle C Schneller A Hartl M Gossenreiter T Homeostatic and Interferon-induced gene expression represent different states of promoter-associated transcription factor ISGF3. bioRxiv [preprint] (2018). 10.1101/377275

[30] Cheon H Stark GR. Unphosphorylated STAT1 prolongs the expression of interferon-induced immune regulatory genes. Proc Natl Acad Sci USA. (2009) 106:9373–78. 10.1073/pnas.0903487106 19478064 PMC2688000

[31] Fink K Grandvaux N. STAT2 and IRF9: Beyond ISGF3. JAK-STAT (2013) 2:e27521. 10.4161/jkst.27521 24498542 PMC3906322

[32] Blaszczyk K Nowicka H Kostyrko K Antonczyk A Wesoly J Bluyssen HAR. The unique role of STAT2 in constitutive and IFN-induced transcription and antiviral responses. Cytokine Growth Factor Rev. (2016) 29:71–81. 10.1016/j.cytogfr.2016.02.010 27053489

[33] Majoros A Platanitis E Kernbauer-Hölzl E Rosebrock F Müller M Decker T. Canonical and non-canonical aspects of JAK–STAT signaling: lessons from interferons for cytokine responses. Front Immunol. (2017) 8:29–41. 10.3389/fimmu.2017.00029 28184222 PMC5266721

[34] Miyamoto M Fujita T Kimura Y Maruyama M Harada H Sudo Y. Regulated expression of a gene encoding a nuclear factor, IRF-1, that specifically binds to IFN-beta gene regulatory elements. Cell (1988) 54:903–13. 3409321 10.1016/s0092-8674(88)91307-4

[35] Goodbourn S Maniatis T. Overlapping positive and negative regulatory domains of the human beta-interferon gene. Proc Nat Acad Sci USA. (1988) 85:1447–51 3422743 10.1073/pnas.85.5.1447 PMC279788

[36] Pine R Decker T Kessler DS Levy DE Darnell JEJ Purification and cloning of interferon-stimulated gene factor 2 (ISGF2): ISGF2 (IRF-1) can bind to the promoters of both beta interferon- and interferon-stimulated genes but is not a primary transcriptional activator of either. Mol Cell Biol. (1990) 10:2448–57. 2342456 10.1128/mcb.10.6.2448-2457.1990 PMC360601

[37] Rengachari S Groiss S Devos JM Caron E Grandvaux N Panne D. Structural basis of STAT2 recognition by IRF9 reveals molecular insights into ISGF3 function. Proc Nat Acad Sci USA. (2018) 115:E601–9. 10.1073/pnas.1718426115 29317535 PMC5789952

[38] Sharf R Meraro D Azriel A Thornton AM Ozato K Petricoin EF. Phosphorylation events modulate the ability of interferon consensus sequence binding protein to interact with interferon regulatory factors and to bind DNA. J Biol Chem. (1997) 272:9785–92. 9092512 10.1074/jbc.272.15.9785

[39] Lin R Hiscott J. A role for casein kinase II phosphorylation in the regulation of IRF-1 transcriptional activity. Mol Cell Biochem. (1999) 191:169–80. 10094406

[40] Ren J Chen X Chen ZJ. IKKβ is an IRF5 kinase that instigates inflammation. Proc Nat Acad Sci USA. (2014) 111:17438–43. 10.1073/pnas.1418516111 25326420 PMC4267374

[41] Fitzgerald KA McWhirter SM Faia KL Rowe DC Latz E Golenbock DT. IKKepsilon and TBK1 are essential components of the IRF3 signaling pathway. Nat Immunol. (2003) 4:491–6. 12692549 10.1038/ni921

[42] Andrilenas KK Ramlall V Kurland J Leung B Harbaugh AG Siggers T. DNA-binding landscape of IRF3, IRF5 and IRF7 dimers: implications for dimer-specific gene regulation. Nucleic Acids Res. (2018) 46:2509–20. 10.1093/nar/gky002 29361124 PMC5861432

[43] Pine R Canova A Schindler C. Tyrosine phosphorylated p91 binds to a single element in the ISGF2/IRF-1 promoter to mediate induction by IFN alpha and IFN gamma, and is likely to autoregulate the p91 gene. EMBO J. (1994) 13:158–67. 8306959 10.1002/j.1460-2075.1994.tb06245.x PMC394789

[44] Pine R Convergence of TNFa and IFNg signalling pathways through synergistic induction of IRF-1/ISGF-2 is mediated by a composite GAS/kB element. Nucleic Acids Res. (1997) 25:4346–54. 9336467 10.1093/nar/25.21.4346 PMC147058

[45] Kanno Y Kozak CA Schindler C Driggers PH Ennist DL Gleason SL. The genomic structure of the murine ICSBP gene reveals the presence of the gamma interferon-responsive element, to which an ISGF3 alpha subunit (or similar) molecule binds. Mol Cell Biol. (1993) 13:3951–63. 8321202 10.1128/mcb.13.7.3951-3963.1993 PMC359937

[46] Contursi C Wang IM Gabriele L Gadina M O'Shea J Morse HC3. IFN consensus sequence binding protein potentiates STAT1-dependent activation of IFNγ-responsive promoters in macrophages. Proc Natl Acad Sci USA. (2000) 97:91–6. 10.1073/pnas.97.1.91 10618376 PMC26621

[47] Mancino A Termanini A Barozzi I Ghisletti S Ostuni R Prosperini E. A dual cis-regulatory code links IRF8 to constitutive and inducible gene expression in macrophages. Genes Dev. (2015) 29:394–408. 10.1101/gad.257592.114 25637355 PMC4335295

[48] Langlais D Barreiro LB Gros P. The macrophage IRF8/IRF1 regulome is required for protection against infections and is associated with chronic inflammation. J Exp Med. (2016) 213:585–603. 10.1084/jem.20151764 27001747 PMC4821649

[49] Farlik M Reutterer B Schindler C Greten F Vogl C Müller M. initiation complex assembly by STAT and NF-kappaB transcription factors regulates nitric oxide synthase expression. Immunity (2010) 33:25–34. 10.1016/j.immuni.2010.07.001 20637660 PMC2914224

[50] Wienerroither S Shukla P Farlik M Majoros A Stych B Vogl C. Cooperative transcriptional activation of antimicrobial genes by STAT and NF-κB pathways by concerted recruitment of the mediator complex. Cell Rep. (2015) 12:300–12. 10.1016/j.celrep.2015.06.021 26146080 PMC4521078

[51] Wienerroither S Rauch I Rosebrock F Jamieson AM Bradner J Muhar M. Regulation of NO synthesis, local inflammation, and innate immunity to pathogens by BET family proteins. Mol Cell Biol. (2014) 34:415–27. 10.1128/MCB.01353-13 24248598 PMC3911514

[52] Ramsauer K Farlik M Zupkovitz G Seiser C Kröger A Hauser H. Distinct modes of action applied by transcription factors STAT1 and IRF1 to initiate transcription of the IFN-gamma-inducible gbp2 gene. Proc Nat Acad Sci USA. (2007) 104:2849–54. 10.1073/pnas.0610944104 17293456 PMC1815270

[53] Leung TH Hoffmann A Baltimore D. One nucleotide in a κB site can determine cofactor specificity for NF-κB dimers. Cell (2004) 118:453–64. 10.1016/j.cell.2004.08.007 15315758

[54] Ogawa S Lozach J Benner C Pascual G Tangirala RK Westin S. Molecular determinants of crosstalk between nuclear receptors and toll-like receptors. Cell (2005) 122:707–21. 10.1016/j.cell.2005.06.029 16143103 PMC1430687

[55] Nan J Wang Y Yang J Stark GR. IRF9 and unphosphorylated STAT2 cooperate with NF-κB to drive IL6 expression. Proc Natl Acad Sci USA. (2018) 115:3906–911. 10.1073/pnas.1714102115 29581268 PMC5899435

[56] Bennett BL Cruz R Lacson RG Manning AM. Interleukin-4 suppression of tumor necrosis factor alpha-stimulated E-selectin gene transcription is mediated by STAT6 antagonism of NF-kappaB. J Biol Chem. (1997) 272:10212–19. 10.1074/jbc.272.15.10212 9092569

[57] Czimmerer Z Daniel B Horvath A Rückerl D Nagy G Kiss M. The transcription factor STAT6 mediates direct repression of inflammatory enhancers and limits activation of alternatively polarized macrophages. Immunity (2018) 48:75–90.e6. 10.1016/j.immuni.2017.12.010 29343442 PMC5772169

[58] Esashi E Wang YH Perng O Qin XF Liu YJ Watowich SS. The signal transducer STAT5 inhibits plasmacytoid dendritic cell development by suppressing transcription factor IRF8. Immunity (2008) 28:509–20. 10.1016/j.immuni.2008.02.013 18342552 PMC2864148

[59] Heinz S Benner C Spann N Bertolino E Yin YC Laslo P. Simple combinations of lineage-determining transcription factors prime cis-regulatory elements required for macrophage and B cell identities. Mol Cell. (2010) 38:576–89. 10.1016/j.molcel.2010.05.004 20513432 PMC2898526

[60] Ghisletti S Barozzi I Mietton F Polletti S De Santa F Venturini E. Identification and characterization of enhancers controlling the inflammatory gene expression program in macrophages. Immunity (2010) 32:317–28. 10.1016/j.immuni.2010.02.008 20206554

[61] Kaikkonen MU Spann NJ Heinz S Romanoski CE Allison KA Stender JD. Remodeling of the enhancer landscape during macrophage activation is coupled to enhancer transcription. Mol Cell (2013) 51:310–25. 10.1016/j.molcel.2013.07.010 23932714 PMC3779836

[62] Kanno Y Levi BZ Tamura T Ozato K. Immune cell-specific amplification of interferon signaling by the IRF-4/8-PU.1 complex. J Interferon Cytokine Res. (2005) 25:770–79. 10.1089/jir.2005.25.770 16375605

[63] Ostuni R Piccolo V Barozzi I Polletti S Termanini A Bonifacio S. Latent enhancers activated by stimulation in differentiated cells. Cell (2013) 152:157–71. 10.1016/j.cell.2012.12.018 23332752

[64] Lawrence T Natoli G. Transcriptional regulation of macrophage polarization: enabling diversity with identity. Nat Rev. (2011) 11:750–61. 10.1038/nri3088 22025054

[65] O'Neill LAJ Pearce EJ. Immunometabolism governs dendritic cell and macrophage function. J Exp Med. (2016) 213:15–23. 10.1084/jem.20151570 26694970 PMC4710204

[66] Schleicher U Paduch K Debus A Obermeyer S König T Kling JC. TNF-mediated restriction of arginase 1 expression in myeloid cells triggers type 2 NO synthase activity at the site of infection. Cell Rep. (2016) 15:1062–75. 10.1016/j.celrep.2016.04.001 27117406 PMC5065922

[67] Van den Bossche J Baardman J Otto NA van der Velden S Neele AE van den Berg SM. Mitochondrial dysfunction prevents repolarization of inflammatory macrophages. Cell Rep. (2016) 17:684–96. 10.1016/j.celrep.2016.09.008 27732846

[68] Pope SD Medzhitov R. Emerging principles of gene expression programs and their regulation. Mol Cell (2018) 71:389–97. 10.1016/j.molcel.2018.07.017 30075140

[69] Ramirez-Carrozzi VR Braas D Bhatt DM Cheng CS Hong C Doty KR. A unifying model for the selective regulation of inducible transcription by CpG islands and nucleosome remodeling. Cell (2009) 138:114–28. 10.1016/j.cell.2009.04.020 19596239 PMC2712736

[70] Hargreaves DC Horng T Medzhitov R. Control of inducible gene expression by signal-dependent transcriptional elongation. Cell (2009) 138:129–45. 10.1016/j.cell.2009.05.047 19596240 PMC2828818

[71] Nicodeme E Jeffrey KL Schaefer U Beinke S Dewell S Chung C-W. Suppression of inflammation by a synthetic histone mimic. Nature (2010) 468:1119–23. 10.1038/nature09589 21068722 PMC5415086

[72] Adelman K Lis JT. Promoter-proximal pausing of RNA polymerase II: emerging roles in metazoans. Nat Rev Genet. (2012) 13:720–31. 10.1038/nrg3293 22986266 PMC3552498

[73] Diamant G Dikstein R. Transcriptional control by NF-κB: elongation in focus. Biochim et Biophys Acta (2013) 1829:937–45. 10.1016/j.bbagrm.2013.04.007 23624258

[74] Hemmi H Takeuchi O Sato S Yamamoto M Kaisho T Sanjo H. The roles of two IkappaB kinase-related kinases in lipopolysaccharide and double stranded RNA signaling and viral infection. J Exp Med. (2004) 199:1641–50. 10.1084/jem.20040520 15210742 PMC2212809

[75] Hoshino K Sugiyama T Matsumoto M Tanaka T Saito M Hemmi H. IκB kinase-α is critical for interferon-α production induced by Toll-like receptors 7 and 9. Nature (2006) 440:949–53. 10.1038/nature04641 16612387

[76] Park S-H Kang K Giannopoulou E Qiao Y Kang K Kim G. Type I interferons and the cytokine TNF cooperatively reprogram the macrophage epigenome to promote inflammatory activation. Nat Immunol. (2017) 18:1104–16. 10.1038/ni.3818 28825701 PMC5605457

[77] Agalioti T Lomvardas S Parekh B Yie J Maniatis T Thanos D. Ordered recruitment of chromatin modifying and general transcription factors to the IFN-beta promoter. Cell (2000) 103:667–78. 10.1016/S0092-8674(00)00169-0 11106736

[78] Panne D Maniatis T Harrison SC. An atomic model of the interferon-beta enhanceosome. Cell (2007) 129:1111–23. 10.1016/j.cell.2007.05.019 17574024 PMC2020837

[79] Freaney JE Kim R Mandhana R Horvath CM. Extensive cooperation of immune master regulators IRF3 and NFκB in RNA Pol II recruitment and pause release in human innate antiviral transcription. Cell Rep. (2013) 4:959–73. 10.1016/j.celrep.2013.07.043 23994473 PMC3792498

[80] Rubio D Xu R-H Remakus S Krouse TE Truckenmiller ME Thapa RJ. Crosstalk between the Type 1 interferon and nuclear factor kappa B pathways confers resistance to a lethal virus infection. Cell Host Microbe (2013) 13:701–10. 10.1016/j.chom.2013.04.015 23768494 PMC3688842

[81] Saliba DG Heger A Eames HL Oikonomopoulos S Teixeira A Blazek K. IRF5:RelA interaction targets inflammatory genes in macrophages. Cell Rep (2014) 8:1308–17. 10.1016/j.celrep.2014.07.034 25159141 PMC4471814

[82] Dalmas E Toubal A Alzaid F Blazek K Eames HL Lebozec K. Irf5 deficiency in macrophages promotes beneficial adipose tissue expansion and insulin sensitivity during obesity. Nat Med. (2015) 21:610–18. 10.1038/nm.3829 25939064

[83] Begitt A Droescher M Meyer T Schmid CD Baker M Antunes F. STAT1-cooperative DNA binding distinguishes type 1 from type 2 interferon signaling. Nat Immunol. (2014) 15:168–76. 10.1038/ni.2794 24413774

[84] Kamijo R Harada H Matsuyama T Bosland M Gerecitano J Shapiro D. Requirement for transcription factor IRF-1 in NO synthase induction in macrophages. Science (1994) 263:1612–15. 7510419 10.1126/science.7510419

[85] Kimura T Kadokawa Y Harada H Matsumoto M Sato M Kashiwazaki Y Essential and non-redundant roles of p48 (ISGF3γ) and IRF-1 in both type I and type II interferon responses, as revealed by gene targeting studies. Genes Cells (1996) 1:115–24. 10.1046/j.1365-2443.1996.08008.x 9078371

[86] Farlik M Rapp B Marie I Levy DE Jamieson AM Decker T. Contribution of a TANK-binding kinase 1-interferon (IFN) regulatory factor 7 pathway to IFN-γ-induced gene expression. Mol Cell Biol. (2012) 32:1032–43. 10.1128/MCB.06021-11 22252317 PMC3295005

[87] Reis LF Ruffner H Stark G Aguet M Weissmann C. Mice devoid of interferon regulatory factor 1 (IRF-1) show normal expression of type I interferon genes. EMBO J. (1994) 13:4798–806. 7957048 10.1002/j.1460-2075.1994.tb06805.x PMC395418

[88] Tailor P Tamura T Morse HC Ozato K. The BXH2 mutation in IRF8 differentially impairs dendritic cell subset development in the mouse. Blood (2008) 111:1942–5. 10.1182/blood-2007-07-100750 18055870 PMC2234043

[89] Shen Y Darnell JE. Antiviral response in cells containing Stat1 with heterologous transactivation domains. J Virol. (2001) 75:2627–33. 10.1128/JVI.75.6.2627-2633.2001 11222685 PMC115886

[90] Kraus TA Lau JF Parisien J-P Horvath CM. A hybrid IRF9-STAT2 protein recapitulates interferon-stimulated gene expression and antiviral response. J Biol Chem. (2003) 278:13033–38. 10.1074/jbc.M212972200 12574168

[91] Ni Z Karaskov E Yu T Callaghan SM Der S Park DS. Apical role for BRG1 in cytokine-induced promoter assembly. Proc Natl Acad Sci USA. (2005) 102:14611–16. 10.1073/pnas.0503070102 16195385 PMC1253546

[92] Mostafavi S Yoshida H Moodley D LeBoité H Rothamel K Raj T. Parsing the interferon transcriptional network and its disease associations. Cell (2016) 164:564–78. 10.1016/j.cell.2015.12.032 26824662 PMC4743492

[93] Au-Yeung N Horvath CM. Histone H2A.Z suppression of interferon-stimulated transcription and antiviral immunity is modulated by GCN5 and BRD2. iScience (2018) 6:68–82. 10.1016/j.isci.2018.07.013 30240626 PMC6137307

[94] Ivashkiv LB IFNγ: signalling, epigenetics and roles in immunity, metabolism, disease and cancer immunotherapy. Nat Rev. (2018) 31:1 10.1038/s41577-018-0029-z PMC6340644 29921905

[95] Kumatori A Yang D Suzuki S Nakamura M. Cooperation of STAT-1 and IRF-1 in interferon-gamma-induced transcription of the gp91(phox) gene. J Biol Chem. (2002) 277:9103–11. 10.1074/jbc.M109803200 11781315

[96] Ni Z Abou El Hassan M Xu Z Yu T Bremner R. The chromatin-remodeling enzyme BRG1 coordinates CIITA induction through many interdependent distal enhancers. Nat Immunol. (2008) 9:785–93. 10.1038/ni.1619 18500344

[97] Hassan El MA Huang K Eswara MBK Xu Z Yu T Aubry A Properties of STAT1 and IRF1 enhancers and the influence of SNPs. BMC Mol Biol. (2017) 18:6 10.1186/s12867-017-0084-1 28274199 PMC5343312

[98] Qiao Y Giannopoulou EG Chan CH Park S-H Gong S Chen J. Synergistic activation of inflammatory cytokine genes by interferon-γ-induced chromatin remodeling and toll-like receptor signaling. Immunity (2013) 39:454–69. 10.1016/j.immuni.2013.08.009 24012417 PMC3857147

[99] Lehtonen A Veckman V Nikula T Lahesmaa R Kinnunen L Matikainen S. Differential expression of IFN regulatory factor 4 gene in human monocyte-derived dendritic cells and macrophages. J Immunol. (2005) 175:6570–9. 10.4049/jimmunol.175.10.6570 16272311

[100] Hsu AT Lupancu TJ Lee M-C Fleetwood AJ Cook AD Hamilton JA. Epigenetic and transcriptional regulation of IL4-induced CCL17 production in human monocytes and murine macrophages. J Biol Chem. (2018) 293: 11415–23. 10.1074/jbc.RA118.002416 29871928 PMC6065189

[101] Satoh T Takeuchi O Vandenbon A Yasuda K Tanaka Y Kumagai Y. The Jmjd3-Irf4 axis regulates M2 macrophage polarization and host responses against helminth infection. Nat Immunol. (2010) 11:936–44. 10.1038/ni.1920 20729857

[102] Piccolo V Curina A Genua M Ghisletti S Simonatto M Sabò A. Opposing macrophage polarization programs show extensive epigenomic and transcriptional cross-talk. Nat Immunol. (2017) 18:530–40. 10.1038/ni.3710 28288101 PMC5524187

[103] Yamagata M Merlie JP Sanes JR. Interspecific comparisons reveal conserved features of the Drosophila Toll protein. Gene (1994) 139:223–8. 8112609 10.1016/0378-1119(94)90760-9

[104] Rosenbauer F Waring JF Foerster J Wietstruk M Philipp D Horak I. Interferon consensus sequence binding protein and interferon regulatory factor-4/pip form a complex that represses the expression of the interferon-stimulated gene-15 in macrophages. Blood (1999) 94:4274–81. 10590072

[105] Meraro D Gleit-Kielmanowicz M Hauser H Levi B-Z. IFN-stimulated gene 15 is synergistically activated through interactions between the myelocyte/lymphocyte-specific transcription factors, PU.1, IFN regulatory factor-8/IFN consensus sequence binding protein, and IFN regulatory factor-4: characterization of a new subtype of IFN-stimulated response element. J Immunol. (2002) 168:6224–31. 10.4049/jimmunol.168.12.6224 12055236

[106] Wu X Briseño CG Durai V Albring JC Haldar M Bagadia P. Mafb lineage tracing to distinguish macrophages from other immune lineages reveals dual identity of Langerhans cells. J Exp Med. (2016) 213:2553–65. 10.1084/jem.20160600 27810926 PMC5110021

[107] Qiao Y Kang K Giannopoulou E Fang C Ivashkiv LB. IFN-γ induces histone 3 lysine 27 trimethylation in a small subset of promoters to stably silence gene expression in human macrophages. Cell Rep. (2016) 16:3121–29. 10.1016/j.celrep.2016.08.051 27653678 PMC5079287

[108] Kang K Park S-H Chen J Qiao Y Giannopoulou E Berg K. Interferon-γ represses M2 gene expression in human macrophages by disassembling enhancers bound by the transcription factor MAF. Immunity (2017) 47:235–250.e4. 10.1016/j.immuni.2017.07.017 28813657 PMC5568089

[109] Biswas SK Lopez-Collazo E. Endotoxin tolerance: new mechanisms, molecules and clinical significance. Trends Immunol. (2009) 30:475–87. 10.1016/j.it.2009.07.009 19781994

[110] Yan Q Carmody RJ Qu Z Ruan Q Jager J Mullican SE. Nuclear factor-κB binding motifs specify Toll-like receptor-induced gene repression through an inducible repressosome. Proc Natl Acad Sci USA. (2012) 109:14140–45. 10.1073/pnas.1119842109 22891325 PMC3435186

[111] Kastenbauer S Ziegler-Heitbrock HW. NF-kappaB1 (p50) is upregulated in lipopolysaccharide tolerance and can block tumor necrosis factor gene expression. Infect Immun. (1999) 67:1553–59. 10084986 10.1128/iai.67.4.1553-1559.1999 PMC96496

[112] Bohuslav J Kravchenko VV Parry GC Erlich JH Gerondakis S Mackman N. Regulation of an essential innate immune response by the p50 subunit of NF-kappaB. J Clin Invest. (1998) 102:1645–52. 10.1172/JCI3877 9802878 PMC509112

[113] Novakovic B Habibi E Wang S-Y Arts RJW Davar R Megchelenbrink W. β-Glucan reverses the epigenetic state of lps-induced immunological tolerance. Cell (2016) 167:1354–1368.e14. 10.1016/j.cell.2016.09.034 27863248 PMC5927328

[114] Chen J Ivashkiv LB. IFN-γ abrogates endotoxin tolerance by facilitating Toll-like receptor-induced chromatin remodeling. Proc Natl Acad Sci USA. (2010) 107:19438–43. 10.1073/pnas.1007816107 20974955 PMC2984206

[115] Foster SL Hargreaves DC Medzhitov R. Gene-specific control of inflammation by TLR-induced chromatin modifications. Nature (2007) 447:972–8. 10.1038/nature05836 17538624

[116] Netea MG Joosten LAB Latz E Mills KHG Natoli G Stunnenberg HG. Trained immunity: a program of innate immune memory in health and disease. Science (2016) 352:aaf1098. 10.1126/science.aaf1098 27102489 PMC5087274

[117] Saeed S Quintin J Kerstens HHD Rao NA Aghajanirefah A Matarese F. Epigenetic programming of monocyte-to-macrophage differentiation and trained innate immunity. Science (2014) 345:1251086. 10.1126/science.1251086 25258085 PMC4242194

[118] Ifrim DC Quintin J Kessel LM Plantinga TS Joosten LAB van der Meer JWM. Defective trained immunity in patients with STAT-1-dependent chronic mucocutaneaous candidiasis. Clin Exp Immunol. (2015) 181:434–40. 10.1111/cei.12642 25880788 PMC4557379

[119] Satpathy AT Wu X Albring JC Murphy KM. Re(de)fining the dendritic cell lineage. Nat Immunol. (2012) 13:1145–54. 10.1038/ni.2467 23160217 PMC3644874

[120] Li HS Watowich SS. A STATus report on DC development. J Leukocyte Biol. (2012) 92:445–59. 10.1189/jlb.0212052 22550127 PMC3427610

[121] Laouar Y Welte T Fu XY Flavell RA. STAT3 is required for Flt3L-dependent dendritic cell differentiation. Immunity (2003) 19:903–912. 10.1016/S1074-7613(03)00332-7 14670306

[122] Liu Y-J. IPC: professional type 1 interferon-producing cells and plasmacytoid dendritic cell precursors. Ann Rev Immunol. (2005) 23:275–306. 10.1146/annurev.immunol.23.021704.115633 15771572

[123] Alculumbre SG Saint-André V Di Domizio J Vargas P Sirven P Bost P. Diversification of human plasmacytoid predendritic cells in response to a single stimulus. Nat Immunol. (2018) 19:63–75. 10.1038/s41590-017-0012-z 29203862

[124] Wimmers F Subedi N van Buuringen N Heister D Vivié J Beeren-Reinieren I. Single-cell analysis reveals that stochasticity and paracrine signaling control interferon-alpha production by plasmacytoid dendritic cells. Nat Commun. (2018) 9:3317. 10.1038/s41467-018-05784-3 30127440 PMC6102223

[125] Luft T Pang KC Thomas E Hertzog P Hart DNJ Trapani J. Type I IFNs enhance the terminal differentiation of dendritic cells. J Immunol. (1998) 161:1947–53. 9712065

[126] Pantel A Teixeira A Haddad E Wood EG Steinman RM Longhi MP Direct Type I IFN but Not MDA5/TLR3 activation of dendritic cells is required for maturation and metabolic shift to glycolysis after poly ic stimulation. PLoS Biol. (2014) 12:e1001759 10.1371/journal.pbio.1001759 24409099 PMC3883643

[127] Hahm B Trifilo MJ Zuniga EI Oldstone MB. Viruses evade the immune system through type I interferon-mediated STAT2-dependent, but STAT1-independent, signaling. Immunity (2005) 22:247–257. 10.1016/j.immuni.2005.01.005 15723812

[128] Li HS Gelbard A Martinez GJ Esashi E Zhang H Nguyen-Jackson H. Cell-intrinsic role for IFN-α-STAT1 signals in regulating murine Peyer patch plasmacytoid dendritic cells and conditioning an inflammatory response. Blood (2011) 118:3879–89. 10.1182/blood-2011-04-349761 21828128 PMC3193265

[129] Geissmann F Gordon S Hume DA Mowat AM Randolph GJ. Unravelling mononuclear phagocyte heterogeneity. Nat Rev. (2010) 10:453–60. 10.1038/nri2784 20467425 PMC3032581

[130] Bedoui S Whitney PG Waithman J Eidsmo L Wakim L Caminschi I. Cross-presentation of viral and self antigens by skin-derived CD103+ dendritic cells. Nat Immunol. (2009) 10:488–95. 10.1038/ni.1724 19349986

[131] Edelson BT KC W Juang R Kohyama M Benoit LA Klekotka PA. Peripheral CD103+ dendritic cells form a unified subset developmentally related to CD8alpha+ conventional dendritic cells. J Exp Med. (2010) 207:823–36. 10.1084/jem.20091627 20351058 PMC2856032

[132] Lindstedt M Lundberg K Borrebaeck CAK. Gene family clustering identifies functionally associated subsets of human *in vivo* blood and tonsillar dendritic cells. J Immunol. (2005) 175:4839–6. 10.4049/jimmunol.175.8.4839 16210585

[133] Robbins SH Walzer T Dembélé D Thibault C Defays A Bessou G. Novel insights into the relationships between dendritic cell subsets in human and mouse revealed by genome-wide expression profiling. Genome Biol. (2008) 9:R17. 10.1186/gb-2008-9-1-r17 18218067 PMC2395256

[134] Ginhoux F Liu K Helft J Bogunovic M Greter M Hashimoto D. The origin and development of nonlymphoid tissue CD103+ DCs. J Exp Med. (2009) 206:3115–30. 10.1084/jem.20091756 20008528 PMC2806447

[135] Durai V Murphy KM. Functions of murine dendritic cells. Immunity (2016) 45:719–36. 10.1016/j.immuni.2016.10.010 27760337 PMC5145312

[136] Garber M Nir Y Goren A Raychowdhury R Thielke A Guttmann M. A high-throughput chromatin immunoprecipitation approach reveals principles of dynamic gene regulation in mammals. a high-throughput chromatin immunoprecipitation approach reveals principles of dynamic gene regulation in mammals. Mol Cell (2012) 47:810–22. 10.1016/j.molcel.2012.07.030 22940246 PMC3873101

[137] Amit I Winter DR Jung S. The role of the local environment and epigenetics in shaping macrophage identity and their effect on tissue homeostasis. Nat Immunol. (2016) 17:18–25. 10.1038/ni.3325 26681458

[138] Dekker J Mirny L. The 3D genome as moderator of chromosomal communication. Cell (2016) 164:1110–21. 10.1016/j.cell.2016.02.007 26967279 PMC4788811

[139] Rozenblatt-Rosen O Stubbington MJT Regev A Teichmann SA. The human cell atlas: from vision to reality. Nat News (2017) 550:451. 10.1038/550451a 29072289

[140] Murata K Wolf M. Cryo-electron microscopy for structural analysis of dynamic biological macromolecules. Biochim Biophys Acta (2018) 1862:324–34. 10.1016/j.bbagen.2017.07.020 28756276

[141] Wang H La Russa M Qi LS. CRISPR/Cas9 in Genome Editing and Beyond. Annu Rev Biochem. (2016) 85:227–64. 10.1146/annurev-biochem-060815-014607 27145843 PMC13384723

